# Methodological approach for allele-specific antibody responses to HEK-293T-based cell lines expressing single MHC class I chain-related gene B antigens

**DOI:** 10.1186/s12860-025-00549-5

**Published:** 2025-07-16

**Authors:** Ji-Ho Jeon, Cheol-Hwa Hong, You-Seok Hyun, Hyeyoung Lee, Eun-Jee Oh, Tai-Gyu Kim, In-Cheol Baek

**Affiliations:** 1 Catholic Hematopoietic Stem Cell Bank, College of Medicine, The Catholic University of Korea, Seoul, Republic of Korea; 2 Department of Microbiology, College of Medicine, The Catholic University of Korea, Seoul, Republic of Korea; 3 Department of Laboratory Medicine, College of Medicine, International St. Mary’s Hospital, Catholic Kwandong University, Incheon, Republic of Korea; 4 Departments of Laboratory Medicine, Seoul St. Mary’s Hospital, College of Medicine, The Catholic University of Korea, Seoul, Republic of Korea; 5 Catholic Hematopoietic Stem Cell Bank, College of Medicine, The Catholic University of Korea, 222, Banpo-daero, Seocho-gu, Seoul, 06591 Republic of Korea

**Keywords:** MHC class I chain-related gene B, MICB antibody, Allotype response, Panel reactive antibody test, Kidney transplant

## Abstract

**Background:**

Antibodies against non-HLA antigens, such as MICA and MICB, have emerged as potential contributors to antibody-mediated rejection and graft failure. While MICA antibodies are well characterized, MICB-specific antibodies remain poorly understood due to the lack of standardized detection tools. To address this gap, we aimed to develop a cell-based platform expressing individual MICB antigens to evaluate the feasibility of detecting allele-specific anti-MICB antibodies in pre-kidney transplant sera.

**Methods:**

*HLA* class I, *MICA*, and *MICB*-null human embryonic kidney (HEK)-293T cells were previously generated by CRISPR/Cas9. We established five cell lines expressing single MICB antigens (each MICB*002, *003, *004, *005:02, and *008 allele). A total of 64 pre-kidney transplant sera were tested to assess anti-MICB antibody responses to the five cell lines using flow cytometry.

**Results:**

We successfully established and validated five HEK-293T cell lines each expressing a single MICB antigen using anti-MICB monoclonal antibody staining. No anti-MICB antibodies were detected in any of the 64 pre-transplant sera tested. This finding may reflect a low incidence of sensitization to MICB in this patient population and suggests the need for larger, more diverse cohorts in future studies to fully assess the prevalence of anti-MICB responses. The established cell lines provide a promising tool for future investigation of allele-specific anti-MICB antibody responses.

**Conclusions:**

While the present study did not detect allele-specific anti-MICB antibody responses, establishing HEK-293T cell lines expressing single MICB antigens represents a significant methodological advance. This platform enables the potential assessment of immune responses targeted to individual MICB allotypes, thus offering new avenues for the future study of MICB immunogenicity in transplantation settings.

**Supplementary Information:**

The online version contains supplementary material available at 10.1186/s12860-025-00549-5.

## Introduction

Pre-transplant cross-match testing, which examines the serum cytotoxicity of the recipient to the lymphocytes of the donor, was presented in early kidney transplantation [1]. Donor human leukocyte antigen-specific antibodies (DSAs) caused late-stage graft loss due to antibody-mediated allograft injury. To ensure successful transplantation and prevent allograft rejection, several techniques have been developed to detect anti-HLA antibodies. These include cell-based and solid-phase methods such as complement-dependent cytotoxicity (CDC), flow cytometry method (FCM), enzyme-linked immunosorbent assay (ELISA), and Luminex-based assay [2, 3].

Recently, the Luminex method has been applied in clinical practice. Recent studies have demonstrated the versatility of the Luminex platform across various clinical applications. For example, during the COVID-19 pandemic, Luminex-based assays were employed to detect antibodies against SARS-CoV-2, showcasing the method’s adaptability to emerging infectious diseases. In these applications, Luminex technology demonstrated high sensitivity and specificity, underscoring its utility in serological surveys and vaccine efficacy studies [4]. Beyond transplantation and infectious disease monitoring, the Luminex method has been applied in pharmacogenomics and personalized medicine. Its ability to simultaneously analyze multiple biomarkers makes it invaluable for drug-induced liver injury (DILI) testing, where rapid and reliable detection of microRNAs and protein biomarkers is essential. The integration of Dynamic Chemical Labeling with the Luminex platform has led to the development of assays capable of direct, multiplex detection, thereby enhancing diagnostic accuracy and patient care [5]. Luminex assays for antibody measurement using beads or recombinant proteins are highly sensitive, raising questions of clinical relevance [6, 7].

The major histocompatibility complex (MHC) region includes various polymorphic genes [8]. A total of 586 MHC class I chain-related gene A (Gene ID: 4276; *MICA*) alleles and 307 MHC class I chain-related gene B (Gene ID: 4277; *MICB*) alleles have been identified in the Immuno Polymorphism Database (IPD)-IMGT/HLA database release version 3.60 in April 2025 (statistics: https://www.ebi.ac.uk/ipd/imgt/hla/about/statistics/, version: https://www.ebi.ac.uk/ipd/imgt/hla/release). Both *MICA* and *MICB* are non-classical MHC class I molecules that are stress-inducible and can be expressed on primary cells and various tumors. MICA and MICB proteins possess α1, α2, and α3 extracellular domains, a transmembrane region, and a cytoplasmic tail. These molecules serve as ligands for the natural killer (NK) group 2D (NKG2D) receptor, mediating immune responses through activation of NK cells, γδ T cells, and some subsets of CD8 + T cells [9–18]. Although *MICA* and *MICB* exhibit high levels of polymorphism among non-classical MHC class I genes—with over 500 and 300 alleles identified, respectively—they are less polymorphic than classical HLA class I molecules, which include more than 10,000 alleles. The polymorphisms, particularly within the extracellular domains of MICA and MICB, influence their binding affinity to the NKG2D receptor and modulate subsequent immune activation (statistics: https://www.ebi.ac.uk/ipd/imgt/hla/about/statistics/) [8]. Exposure to mismatched MICA or MICB antigens following transplantation may lead to the development of anti-MICA or anti-MICB antibodies, which have been implicated in antibody-mediated rejection and graft failure. Recent studies have emphasized the clinical importance of detecting anti-MICA antibodies in transplant recipients [19–21].

However, there are no commercially available panel reactive antibody (PRA) test kits for the MICB antibodies. Eleven *MICA* alleles were transduced into each *HLA* class I, *MICA*, and *MICB*-null human embryonic kidney (HEK)-293T cell. The reactivities between the MICA antigens and the pre-kidney transplantation sera were confirmed using established cell lines [22, 23].

The primary objective of the study was to demonstrate the feasibility of recombinant cell lines as a test platform for anti-MICB antibody detection. To address the need for allele-specific detection of anti-MICB antibodies, we established HEK-293T cell lines expressing five individual MICB antigens, corresponding to the most frequently observed alleles in the Korean population.

## Materials and methods

### Cell culture

In previous studies, the H1E-25 HEK-293T cell line, which had HLA class I deleted using CRISPR/Cas9, was selected to rule out alloantibody responses to HLA [22]. Additionally, the H1ME-5 HEK-293T cell line was constructed not to express both MICA and MICB proteins [23]. These cell lines (H1E-25 and H1ME-5) were not commercially obtained or sourced from external laboratories, but were established and validated in our previous studies [22, 23], and used here for continuity and methodological consistency. Cell lines deficient in *HLA* class I, *MICA*, and *MICB* genes were generated by sequential CRISPR/Cas9-mediated knockouts. These cell lines were used as internal negative controls to assess background binding in flow cytometry assays. *MICB* alleles were identified using sequence-based typing [24]. *HLA* class I, *MICA* and *MICB* null-293T (H1ME-5) cell lines were cultured in Dulbecco’s modified Eagle’s medium (Lonza, Walkersville, MD, USA) supplemented with 1% L-glutamine (Lonza), 1% penicillin–streptomycin (Lonza), and 10% fetal bovine serum (Hyclone, Logan, UT, USA). After we thawed the frozen cell lines, expression of MICB was verified using flow cytometry. We used the cells within a month and froze them in a medium consisting of DMSO, DMEM, and FBS in a 1:4:5 ratio [22, 23, 25, 26].

### Sera of pre-kidney transplantation

We used sera from 64 pre-kidney transplantation patients, obtained during a previous study [23]. Briefly, pre-kidney transplant sera were obtained to be requested for the PRA testing of HLA and MICA at the Department of Laboratory Medicine, St. Mary’s Hospital in Seoul from January 2014 to December 2016. Samples were serialized and stored at − 20 °C. We used samples that were thawed and vortexed at 25 °C. All subjects provided informed consent to participate in the study. Furthermore, written informed consent was obtained from each participant and their parents or guardians. The study protocol was approved by the Institutional Review Board (IRB) of the Catholic University of Korea (IRB Number: MC13SISI0126 [DNA], MC19SNSI0068 [serum]), Seoul, Korea, and the study were conducted in accordance with the Declaration of Helsinki.

### Production of lentiviruses expressing single MICB antigens

We produced lentiviruses expressing single *MICB* antigens using the method described in a previous study [23]. Briefly, RNA isolation of peripheral blood mononuclear cells was performed using NucleoSpin^®^ RNA (Macherey-Nagel, GmbH, Duren, Germany) following the manufacturer’s protocol. We synthesized cDNA using the SuperiorScript III cDNA Synthesis kit (Enzynomics, Daejeon, Korea). Each *MICB* allele was amplified using forward (5′-UTR) and reverse (3′-UTR) primers: 5′-TCCCGGCCCTTCCGGACCACTGCTGAGCAGCTGAGAA-3′ (forward) and 5′- GAGGTTGATTGTCGACTTAGATTCAGCTAGTTGAATCCTGGCCGCCTGGCT-3′ (reverse). PCR was carried out in 30-µL reaction mixtures, containing 50 ng cDNA, 10 µM primer sets, 0.6 µL of each dNTP (25 mM), 10× PCR buffer (Kapa Biosystem, Wilmington, MA, USA), distilled water, and 1.5 U of Taq DNA polymerase (Kapa Biosystem) in 200-µL PCR tubes (Axygen, Hangzhou, China). We performed the cloning into the pCDH vector (#CD523A-1; SBI, Palo Alto, CA, USA) that copGFP was removed by the restriction enzymes Sal1 (R009S; Enzynomics) and BspE1 (R108S; Enzynomics) using EZ-Fusion Cloning kit (EZ015M; Enzynomics). Plasmids were confirmed using Sanger sequencing. 5 × 10^6^ HEK-293T cells were seeded to produce lentiviruses encoding each molecule (MICB*002, *003, *004, *005:02, and *008) in T75 flasks. After 24 h, we co-transfected 10 µg of the cloned *MICB* pCDH plasmid and lentivirus packaging plasmids (5 µg pMD2.G and 5 µg psPAX2, cat nos. #12259, #12260; Addgene, Cambridge, MA, USA) into HEK-293T cells using the Lipofectamine reagent (Invitrogen, Carlsbad, CA, USA). After transfection for 48 h, the supernatants of the lentivirus were harvested. We filtered the supernatants using 0.45-µm filters.

### Generation of cell lines expressing single MICB antigens

Cell lines expressing single MICB antigens were generated as following the previous study [23]. Briefly, we seeded 5 × 10^5^ 293T cells/mL into each well of 6-well plates for the transduction of each lentivirus. After 24 h, we added 8 µg/mL of polybrene and 500 µL of the lentiviral supernatant to the 293T cell cultures. After transduction for 48 h, we cultured the cells using anti-MICB-APC (FAB1599A; R&D Systems) and analyzed using FCM. After harvesting, the cells were fluorescently stained by labeled anti-human MICB antibodies for 30 min at 4 °C in the dark. They were analyzed using a Fortessa flow cytometer or FACS Canto (BD Biosciences, San Jose, CA, USA). The single allele cell lines were grown up and used as stable cell lines.

### Response measurement of anti-MICB antibodies in Sera of pre-kidney transplantation by FCM

Testing for anti-MICB antibodies detection was performed in sera of pre-kidney transplantation to the single MICB-expressing cell lines by FCM as following the previous study [23]. Briefly, 20 µL of the single MICB-expressing cell lines adjusted to 2 × 10^5^ cells/mL and 5 µL of the serum was incubated using anti-human IgG Fc-FITC (F031501; DAKO, Tokyo, Japan) at 25 °C for 30 min. After washing twice, 2 µL of anti-human IgG (FITC-F (ab′) 2 anti-human IgG, DAKO) was cross-reacted in a dark room for 30 min. After washing three times, the cell suspension was processed by adding 500 µL of PBS. Fluorescence response of the cells was determined using FCM with FACS Fortessa or Canto. The threshold of anti-MICB positivity was defined as a higher MFI value than H1ME-5.

## Results

### Establishment of MICB cell lines expressing single MICB antigens

**Fig. 1:**
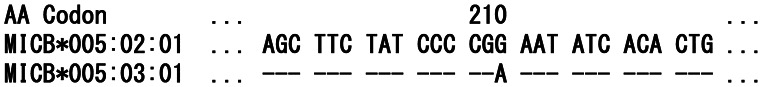
Alignment of MICB*005:02:01 and *005:03:01 in the IMGT/HLA database (https://www.ebi.ac.uk/cgi-bin/ipd/pl/align.cgi). A single nucleotide changes at codon 210 (CGG in *005:02:01 vs. CGA in *005:03:01) results in the same amino acid, arginine (Arg). Hence, we excluded MICB*005:03 allele from the establishment of single MICB-expressing cell lines

Cell lines expressing five single MICB antigens were established from the H1ME-5 HEK-293T cell line. We performed the transduction and the cloning using lentiviral vectors expressing each MICB antigen, including MICB*002, *003, *004, *005:02, and *008. The frequencies of the alleles were over 3% in South Koreans. However, the frequency of MICB*005:03 allele (0.9–8.3%) was excluded to establish cell lines expressing single MICB antigens because it did not cause a new phenotype compared to MICB*005:02 and therefore would not have resulted in more diversity in the cell line panel. In detail, based on alignment of MICB*005:02:01 and *005:03:01 in the IMGT/HLA database, a single nucleotide sequence difference in codon 210 (CGG and CGA, respectively) encodes the same amino acid, arginine (Arg) (Fig. 1). The five major alleles have been distributed in Central Chinese, Zhejiang Han Chinese, Thai, Welsh, Spanish, and German populations, including our previous study, so we have set the standard (Table 1) [24, 27–33].

**Table 1:** *MICB* allele frequencies

Population	Allele frequencies (%)								FCM [23]
	Korean [24]	Korean [27]	Central Chinese [28]	Zhejiang Han Chinese [29]	Thai [30]	Welsh [31]	Spanish [32]	German [33]	
Total number (2*n*)	400	278	426	800	200	332	200	2,403,792	
MICB*002	13.5	11.5	12.4^a^	12.3	36.0***	15.7	17	18.9	v
MICB*003	3.8	2.5	10.1	1.8	0.5	4.8	3.5	—	v
MICB*004	11.5	8.3	16.9^a^	8.4	12.3	25.6*	13.5	21.7^c^	v
MICB*005:01	—	—	—		0.0	—	1.0	—	
MICB*005:02	55.5	57.2	44.4^a^	57.5^b^	38.4*	35.5**	48.0	43.9^c^	v
MICB*005:03	5.3	8.3	—	5.6	3.0	0.9	4.5	—	
MICB*008	5.3	6.8	8.9	7.9	8.9	15.4*	12.5	11	v
MICB*009N	2.8	2.2	7.3	0.9	—	0.0	0.0	0.0	
MICB*013	—	—	—	0.1	2.0	0.3	—	1.4	
MICB*014	2	3.2	—	3.9	3.6	1.8	0.0	2.2	
MICB*018	0.3	—	—	0.1	—	—	—	—	
MICB*019	0.3	—	—	0.3	—	—	—	—	

*MICB*, major histocompatibility complex class I chain-related gene B; FCM, flow cytometric method [23]

a, MICB*002 is displayed including MICB*014, and MICB*005:02 is displayed including MICB*00503, *010, and *013

b, MICB*005:02 is displayed including MICB*010

c, MICB*004 is displayed including MICB*028, and MICB*005:02 is displayed including MICB*003, *005:01, *005:03, and *010

* *P* < 0.05, **P < 0.01, and ***P < 0.001 compared with the Korean population

### Responses of established cell lines expressing single MICB antigens and Sera of pre-kidney transplantation by FCM

**Fig. 2:**
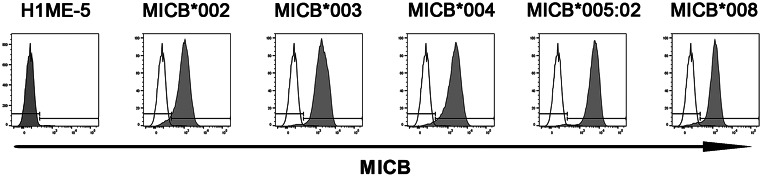
Establishment of cell lines expressing the five single MICB antigens from the H1ME-5 HEK-293T cell line. Transduction and cloning using lentivirus vectors expressing each *MICB* allele, including MICB*002, *003, *004, *005:02, and *008. The MICB molecules expressed on the surface of selected cells were verified using anti-MICB APC (gray) antibodies

**Fig. 3:**
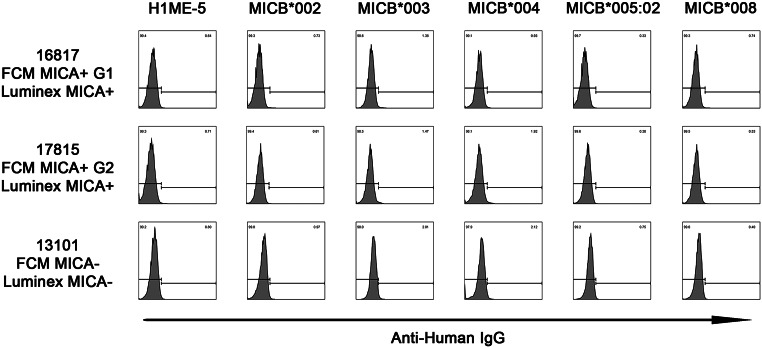
Representative response patterns of single MICB-expressing cell lines to the sera that MICA antigens were confirmed as negative by FCM and Luminex method. FCM MICA + G1, G2, and MICA- showed response patterns of single MICA-expressing cell lines in the previous study [23]. FCM MICA + G1 and G2 were each one of the two positive (Sample ID: 16817 and 17815) serum confirmed by the Luminex method. FCM MICA- was a negative (Sample ID: 13101) serum confirmed by the Luminex method in the previous study [23]

After transduction, we sorted and cultured the MICB-positive cells on day 6. The high expression of five single MICB antigens was demonstrated by the fluorescence-activated cell sorting (FACS) analysis using monoclonal anti-MICB antibodies (Fig. 2). Representative responses of established cell lines expressing single MICB antigens to anti-MICA positive sera verified using FCM or the Luminex method are shown as negative in Fig. 3. FCM MICA + G1, G2, and MICA − showed response patterns of established cell lines expressing single MICA antigens in the previous study [23]. FCM MICA PRA tests showed positive results in antigenic cell lines in two samples, G1 was defined as group 1 in the previous study because the FCM MICA PRA test for Sample ID 16,817 showed positive reactions in the antigenic cell lines. G2 was defined as group 2 in the present study because the FCM MICA PRA test for Sample ID 17,815 showed a different pattern of positive reactions in the antigenic cell lines when compared to G1 in the previous study. FCM MICA + G1 and G2 were each one of the two positive (Sample ID: 16817 and 17815) serum samples using the Luminex method. FCM MICA − was negative (Sample ID: 13101) serum, as determined by using the Luminex method.

MICB antigen–antibody reactivity was analyzed in the sera of 64 patients with renal transplants (Fig. S1). None of the pre-transplant sera tested responded to the MICB-expressing cell lines. Based on prior studies reporting detectable anti-MICA antibodies in pre-transplant patients [23], we had anticipated that anti-MICB antibodies might also be present, considering the structural similarities between MICA and MICB. However, contrary to expectations, no anti-MICB reactivity was observed in this cohort (Fig. S1).

Flow cytometric analysis confirmed that sera from pre-transplant patients that did not react with any MICB-expressing cell lines also showed no non-specific binding to HLA class I/MICA/MICB triple-knockout cell lines (H1ME-5, Fig. 2), supporting their suitability as negative sera controls within this study.

## Discussion

To date, human cell line-based studies on MICB antibody responses in kidney transplant patients have not been conducted. The observation of the response of MICB molecules to renal transplant biopsy, in 2006, is the latest data on the direct observation of the MICB antigen in renal transplant patients [19]. MICB expression caused rejection of allograft kidneys and pancreas. However, this was an observation of the MICB molecule, not a detection of the MICB antibody using the MICB antigen [34]. In 2013, a positive XM-ONE assay (AbSorber AB, Stockholm, Sweden), a commercial flow cytometry-based assay designed to detect donor-specific anti-endothelial cell antibodies, confirmed the presence or absence of HLA and MICA DSAs in all patients. However, this was not done for MICB [35]. A study suggests that the antigens expressed on the surfaces of endothelial cells and not found on peripheral blood lymphocytes include specific endothelium-expressed autoantigens and polymorphic antigen families that induce the production of antibodies distinct from HLA. However, antibodies against MICA are associated with allograft rejection, and antibodies against MICB are rarely found in these patients [36]. The frequency of MIC antibodies in patients with rejected renal transplants without HLA antibodies was assessed using MICA*001, *002, *007, *008, and MICB*002 antigens produced in Escherichia coli and immobilized on ELISA plates. In normal patients, the frequency of positive samples was 3% (1 in 35) for the MICB*002 antibody. Among the patients with graft rejections, the MICB*002 antibody was more positive than among the normal patients [37].

The five major alleles are distributed throughout the worldwide population and Korean individuals has also been well investigated [24, 27]. In the present study, the *MICB* alleles having the frequency of > 3% in Korean individuals were selected to produce cell lines expressing single MICB antigens for FCM. In the case of MICA study, antibody responses to MICA antigens result from cross-reactions by sharing a common epitope [23]. The 64 sera collected from patients with kidney pre-transplants showed no response to five single MICB-expressing cell lines (Fig. 3 and Fig. S1). This result may primarily reflect the absence of anti-MICB antibodies in the pre-transplant sera. Given that high surface expression of MICB was confirmed in all five cell lines using monoclonal antibody-based flow cytometry, the robustness of the antigen presentation system supports the reliability of this negative finding. However, the possibility of conformational differences affecting antibody recognition cannot be completely excluded. Occasional weak background signals were observed, which are likely attributable to non-specific interactions with other cell surface molecules unrelated to MIC antigens.

Future studies are warranted to validate antigen expression across different MIC molecules more precisely. For instance, proteolytic shedding of MICB from the tumor cell surface can reduce detectable levels, as tumor cells often shed MICA/B proteins to evade immune detection [38]. The specificity and affinity of the antibodies used are critical. Antibodies targeting the α3 domain of MICB have been shown to prevent shedding and enhance detection. Utilizing antibodies that do not effectively bind to this domain may result in diminished reactivity [38]. Variations in MICB expression among different patient populations can influence reactivity outcomes. Factors such as genetic polymorphisms, tumor type, and disease stage can lead to heterogeneous MICB expression. Studies have demonstrated that MICA/B expression is not constitutive in healthy tissues but is upregulated in various tumor types, with expression levels varying across different cancers [39]. Addressing these factors involves optimizing detection methods to enhance sensitivity, selecting antibodies with high specificity for critical MICB domains, and accounting for patient-specific variations in MICB expression. Such considerations are essential for accurately interpreting negative reactivity results in MICB-related studies.

MICB, a stress-induced ligand expressed on endothelial cells, can become a target for the recipient’s immune system post-transplantation. Antibodies directed against MICA and/or MICB have been implicated in antibody-mediated rejection; however, earlier studies used reagents that did not distinguish between MICA and MICB antigens. This is particularly significant in cases where HLA compatibility is ensured, yet rejection occurs, suggesting that non-HLA antibodies, such as those against MICB, play a critical role [40].

Recognizing the role of MICBs in transplant rejection underscores the need for further research into non-HLA antibodies. Future research should focus on: (1) Revealing the pathways by which anti-MICB antibodies mediate graft damage may shed light on new therapeutic objectives. (2) Determining the prevalence of anti-MICB antibodies in diverse populations may help classify risks and guide clinical decision-making. (3) Clinical trials evaluating treatments specifically addressing MICB-associated immune responses may help improve outcomes in transplant recipients. In summary, acknowledging and investigating the clinical implications of MICB responsiveness is essential for enhancing transplant success and shaping future research directions in transplant immunology.

This study had several limitations, including the lack of IgM, no measurement of IgG subclasses (which is inaccurate since there are no pan-IgG positive samples anyway), lack of adequate serum positive controls, lack of threshold definition of anti-MICB positivity, and a limitation to a cohort of less promising pre-transplant sera (less promising compared to post-transplant sera). It might have been more informative to analyze post-transplant sera from patients who underwent an indication biopsy (typically for rejection) rather than examining pre-transplant sera, where the prevalence of anti-MICB antibodies may be lower. There is no negative control group, typically represented by healthy individual. These results suggest that further studies incorporating larger cohorts, healthy controls, and patients with varying clinical outcomes (e.g., rejection and non-rejection) are necessary to validate and expand upon these observations. Another limitation of this study is the lack of direct quantitative comparison of MICA and MICB expression levels using a cross-reactive monoclonal antibody targeting shared epitopes. Due to amount of available samples, simultaneous testing of both MICA and MICB was not conducted in this study.

## Conclusion

No allele-specific anti-MICB antibody response has been detected in the pre-kidney transplant sera. Establishing HEK-293T cell lines expressing a single MICB antigen may help to present an important methodological approach. This platform enables the potential assessment of immune responses targeting individual MICB allotypes. Furthermore, it may provide new avenues for future MICB immunogenicity studies in transplant settings.

## Electronic supplementary material

Below is the link to the electronic supplementary material.

Supplementary Material 1

## Data Availability

The datasets for the PCR primers generated and/or analysed during the current study are available in the NCBI/Gene repository, https://www.ncbi.nlm.nih.gov/gene/4277. All data generated or analyzed during this study are included in this published article.
