# Supplementary material for: Methodological approach for allele-specific antibody responses to HEK-293T-based cell lines expressing single MHC class I chain-related gene B antigens
Source: BMC Mol Cell Biol. 2025 Jul 16;26:22. doi: 10.1186/s12860-025-00549-5 (PMC12265220; doi:10.1186/s12860-025-00549-5)
Supplement: Supplementary file 1 — Supplementary Material 1 [file 12860_2025_549_MOESM1_ESM.pptx]

## Slide 1
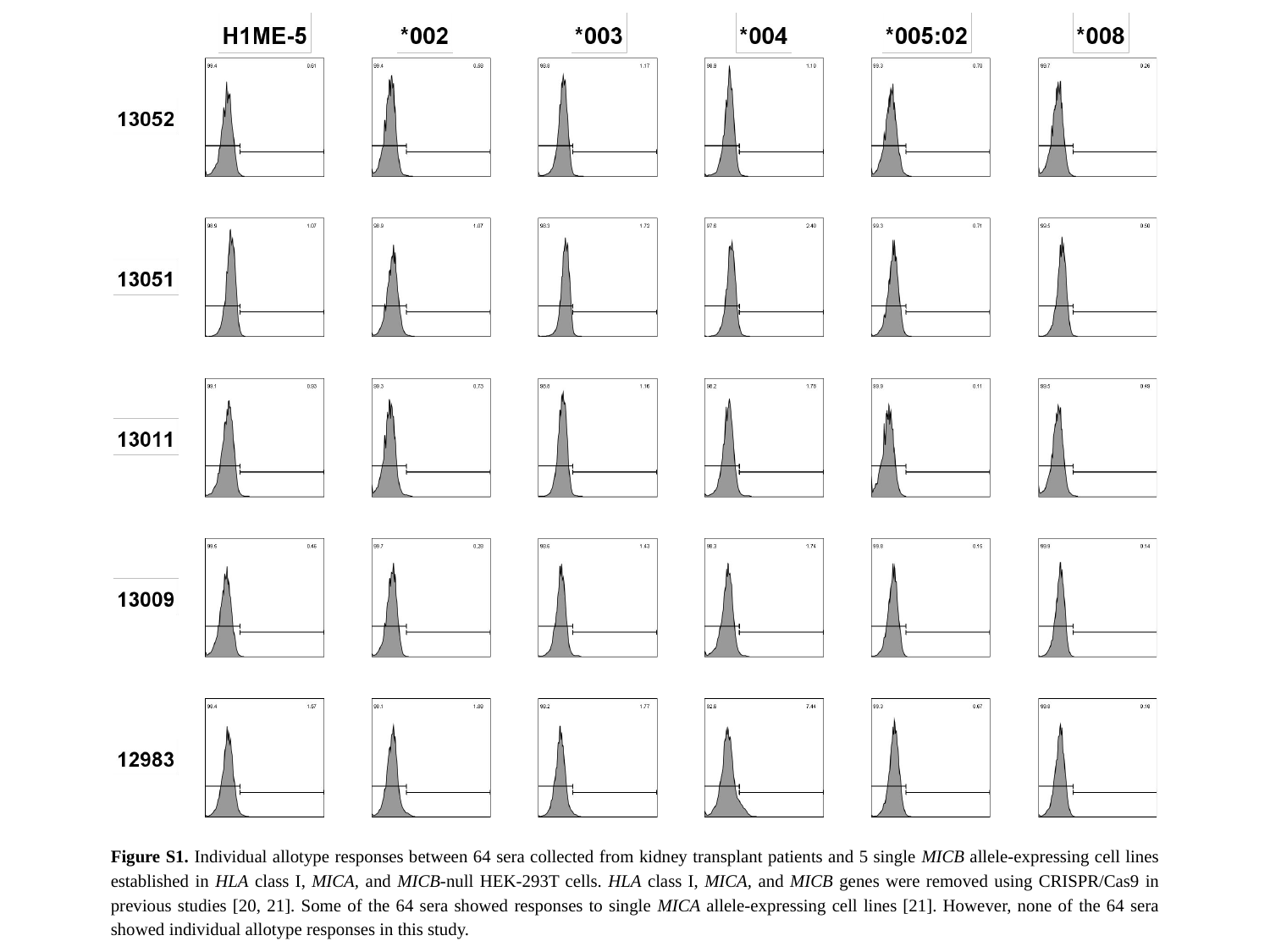

Figure S1. Individual allotype responses between 64 sera collected from kidney transplant patients and 5 single MICB allele-expressing cell lines established in HLA class I, MICA, and MICB-null HEK-293T cells. HLA class I, MICA, and MICB genes were removed using CRISPR/Cas9 in previous studies [20, 21]. Some of the 64 sera showed responses to single MICA allele-expressing cell lines [21]. However, none of the 64 sera showed individual allotype responses in this study.

## Slide 2
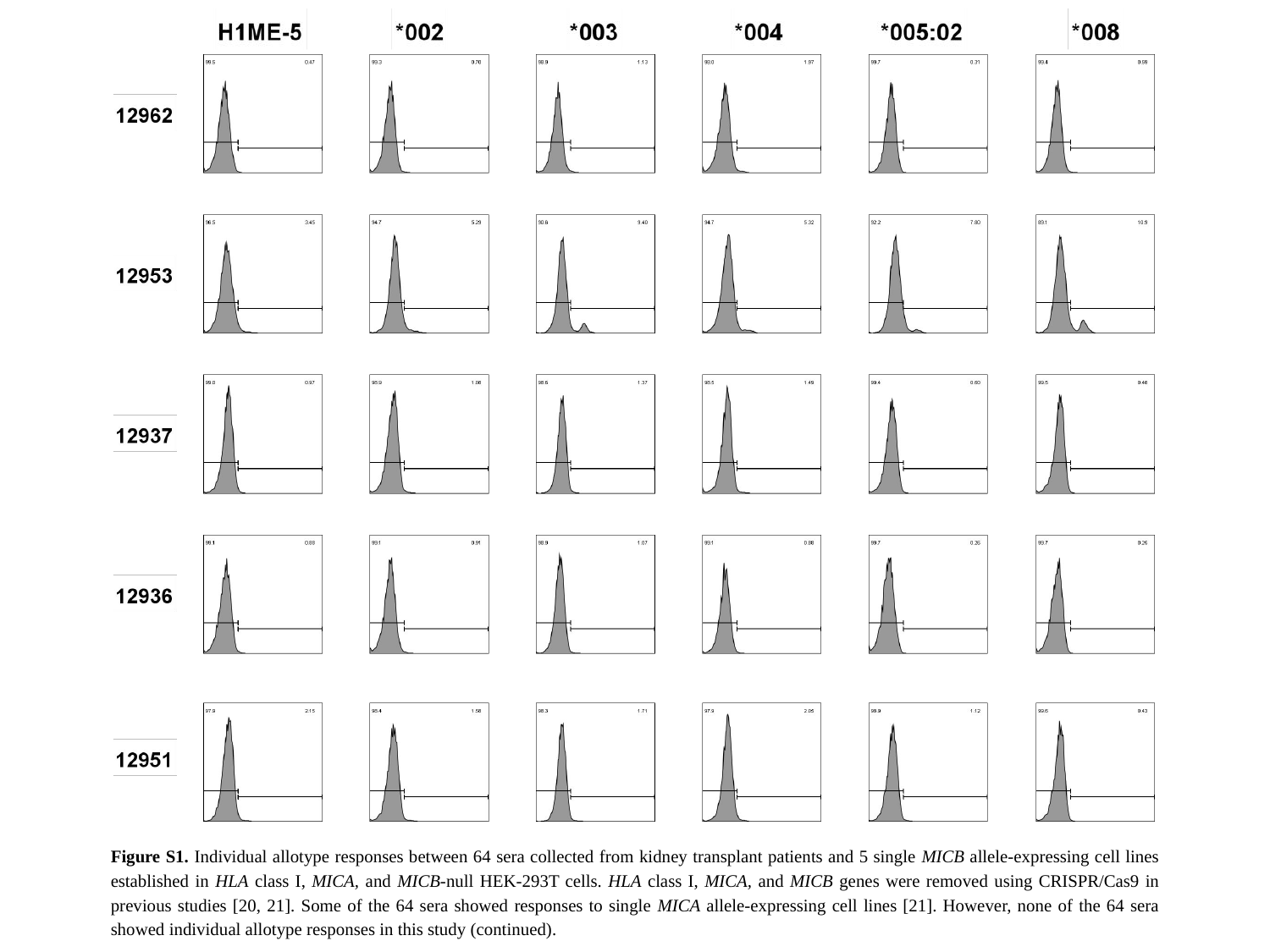

Figure S1. Individual allotype responses between 64 sera collected from kidney transplant patients and 5 single MICB allele-expressing cell lines established in HLA class I, MICA, and MICB-null HEK-293T cells. HLA class I, MICA, and MICB genes were removed using CRISPR/Cas9 in previous studies [20, 21]. Some of the 64 sera showed responses to single MICA allele-expressing cell lines [21]. However, none of the 64 sera showed individual allotype responses in this study (continued).

## Slide 3
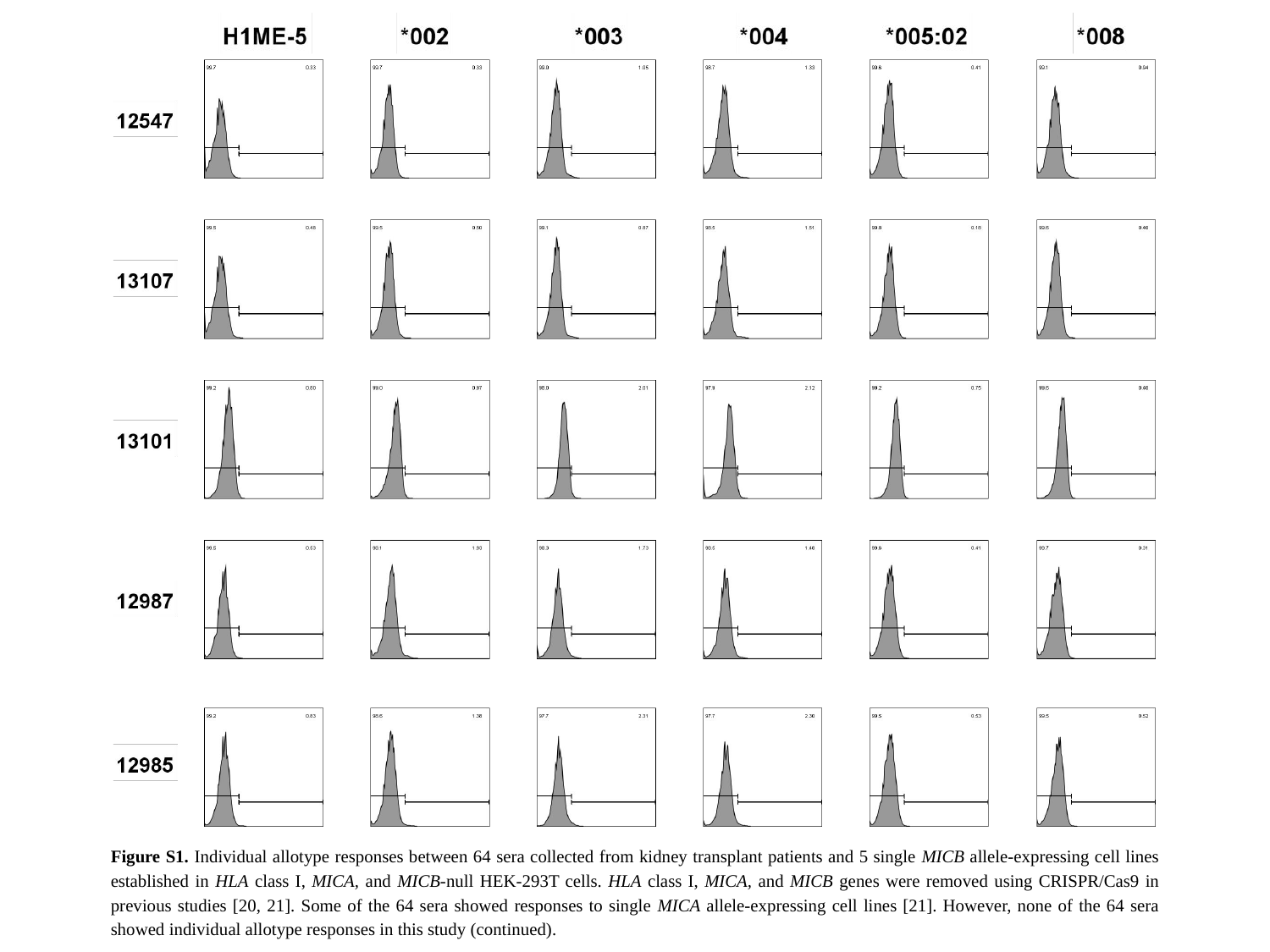

Figure S1. Individual allotype responses between 64 sera collected from kidney transplant patients and 5 single MICB allele-expressing cell lines established in HLA class I, MICA, and MICB-null HEK-293T cells. HLA class I, MICA, and MICB genes were removed using CRISPR/Cas9 in previous studies [20, 21]. Some of the 64 sera showed responses to single MICA allele-expressing cell lines [21]. However, none of the 64 sera showed individual allotype responses in this study (continued).

## Slide 4
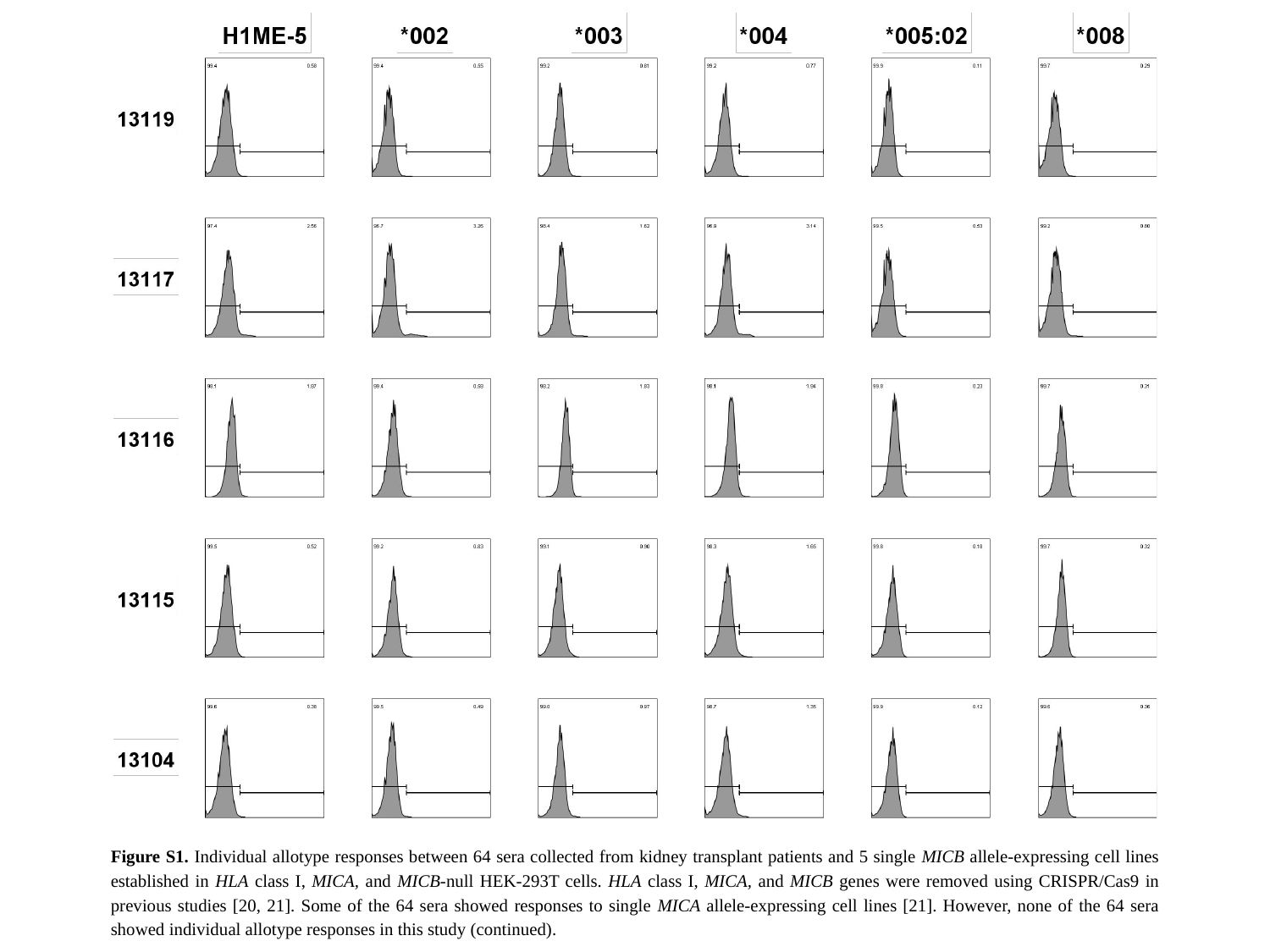

Figure S1. Individual allotype responses between 64 sera collected from kidney transplant patients and 5 single MICB allele-expressing cell lines established in HLA class I, MICA, and MICB-null HEK-293T cells. HLA class I, MICA, and MICB genes were removed using CRISPR/Cas9 in previous studies [20, 21]. Some of the 64 sera showed responses to single MICA allele-expressing cell lines [21]. However, none of the 64 sera showed individual allotype responses in this study (continued).

## Slide 5
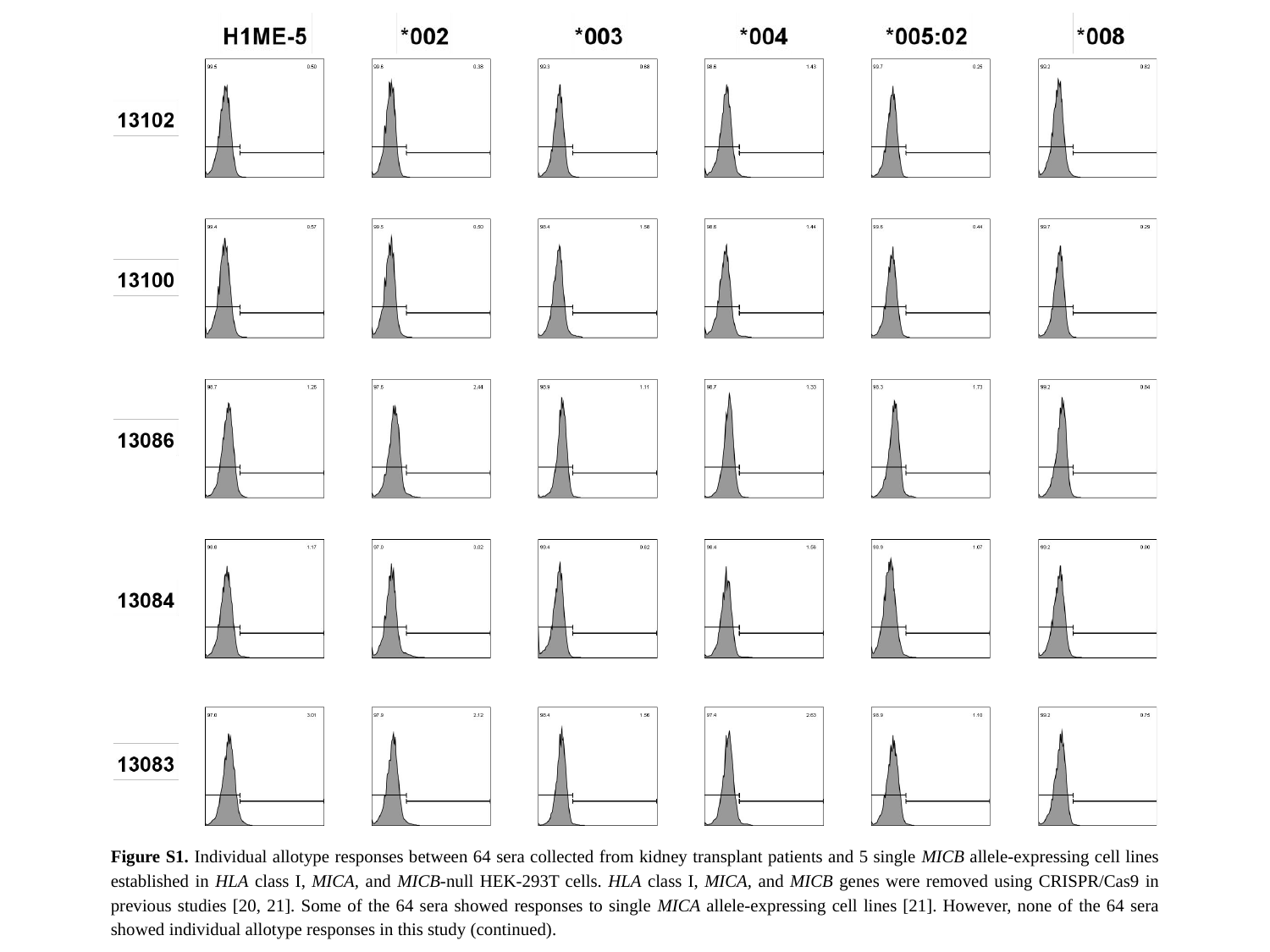

Figure S1. Individual allotype responses between 64 sera collected from kidney transplant patients and 5 single MICB allele-expressing cell lines established in HLA class I, MICA, and MICB-null HEK-293T cells. HLA class I, MICA, and MICB genes were removed using CRISPR/Cas9 in previous studies [20, 21]. Some of the 64 sera showed responses to single MICA allele-expressing cell lines [21]. However, none of the 64 sera showed individual allotype responses in this study (continued).

## Slide 6
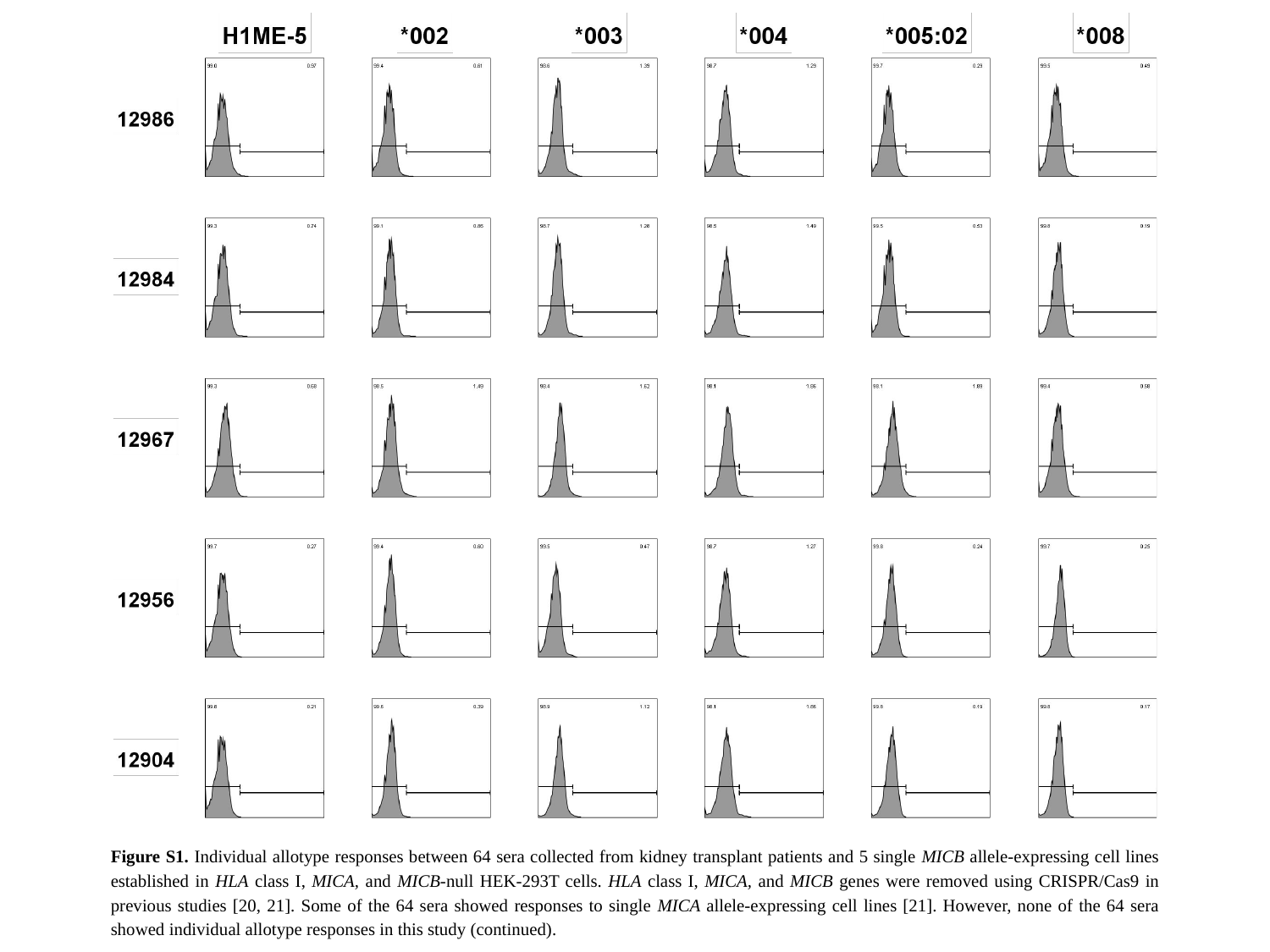

Figure S1. Individual allotype responses between 64 sera collected from kidney transplant patients and 5 single MICB allele-expressing cell lines established in HLA class I, MICA, and MICB-null HEK-293T cells. HLA class I, MICA, and MICB genes were removed using CRISPR/Cas9 in previous studies [20, 21]. Some of the 64 sera showed responses to single MICA allele-expressing cell lines [21]. However, none of the 64 sera showed individual allotype responses in this study (continued).

## Slide 7
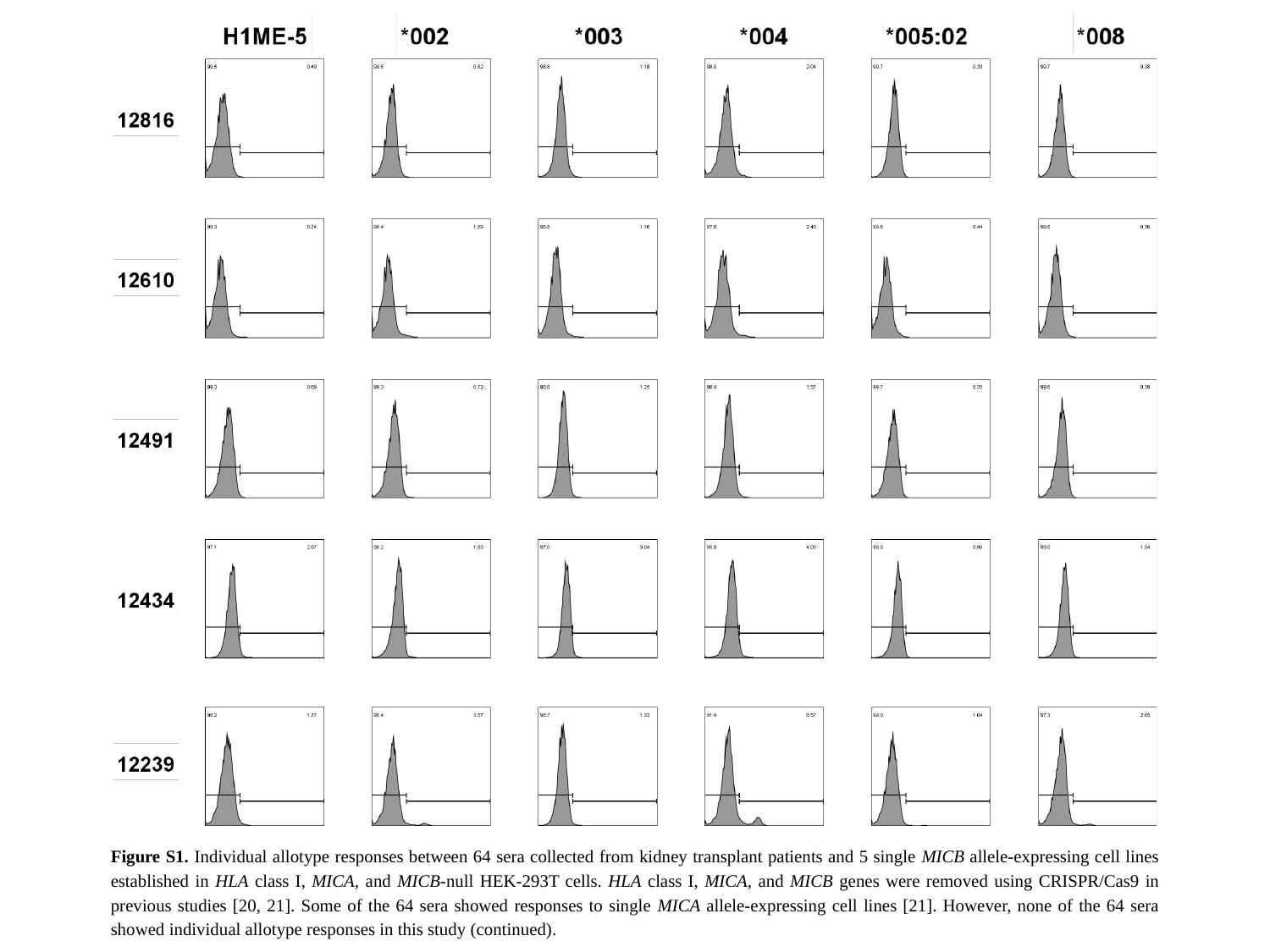

Figure S1. Individual allotype responses between 64 sera collected from kidney transplant patients and 5 single MICB allele-expressing cell lines established in HLA class I, MICA, and MICB-null HEK-293T cells. HLA class I, MICA, and MICB genes were removed using CRISPR/Cas9 in previous studies [20, 21]. Some of the 64 sera showed responses to single MICA allele-expressing cell lines [21]. However, none of the 64 sera showed individual allotype responses in this study (continued).

## Slide 8
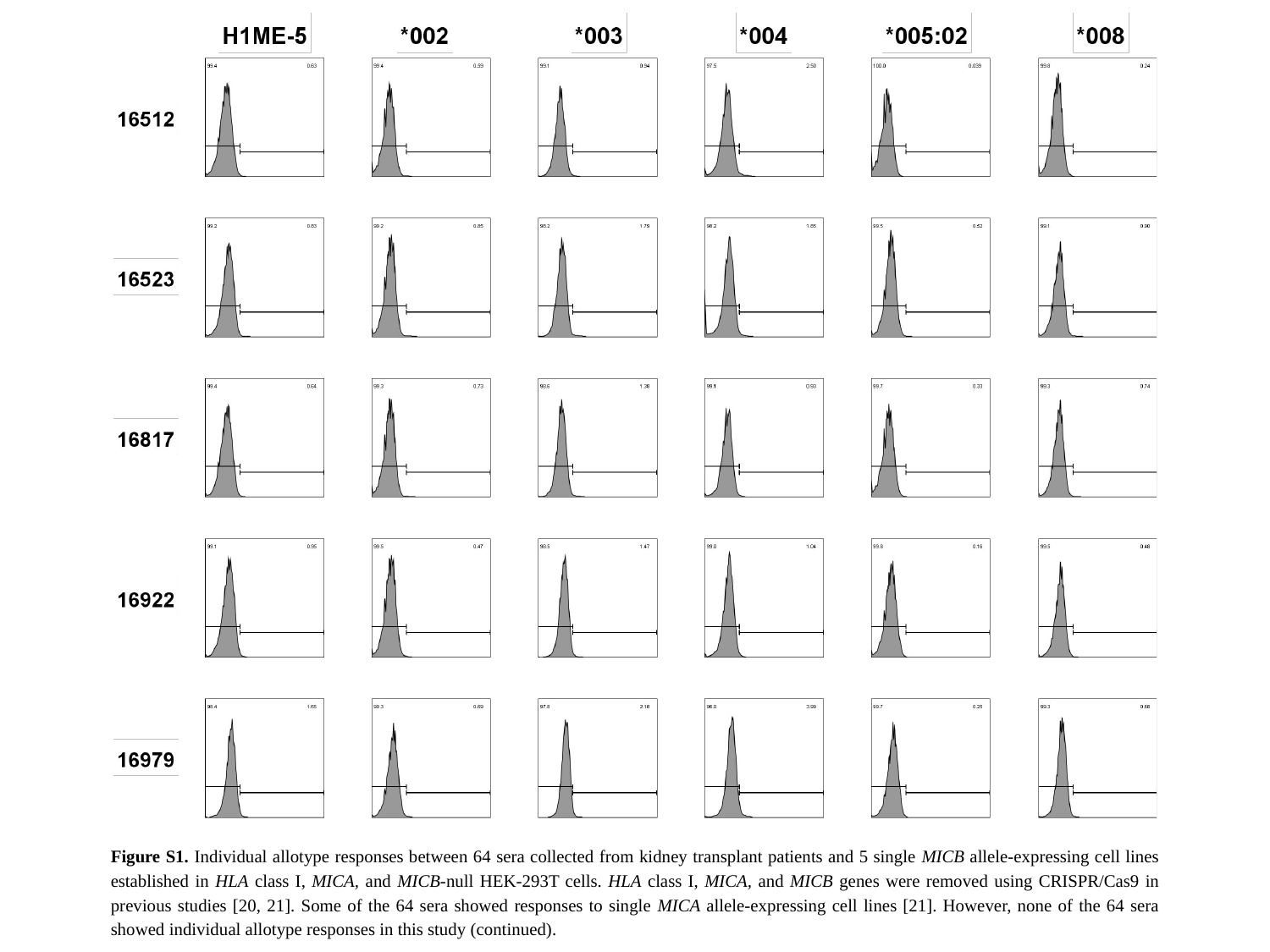

Figure S1. Individual allotype responses between 64 sera collected from kidney transplant patients and 5 single MICB allele-expressing cell lines established in HLA class I, MICA, and MICB-null HEK-293T cells. HLA class I, MICA, and MICB genes were removed using CRISPR/Cas9 in previous studies [20, 21]. Some of the 64 sera showed responses to single MICA allele-expressing cell lines [21]. However, none of the 64 sera showed individual allotype responses in this study (continued).

## Slide 9
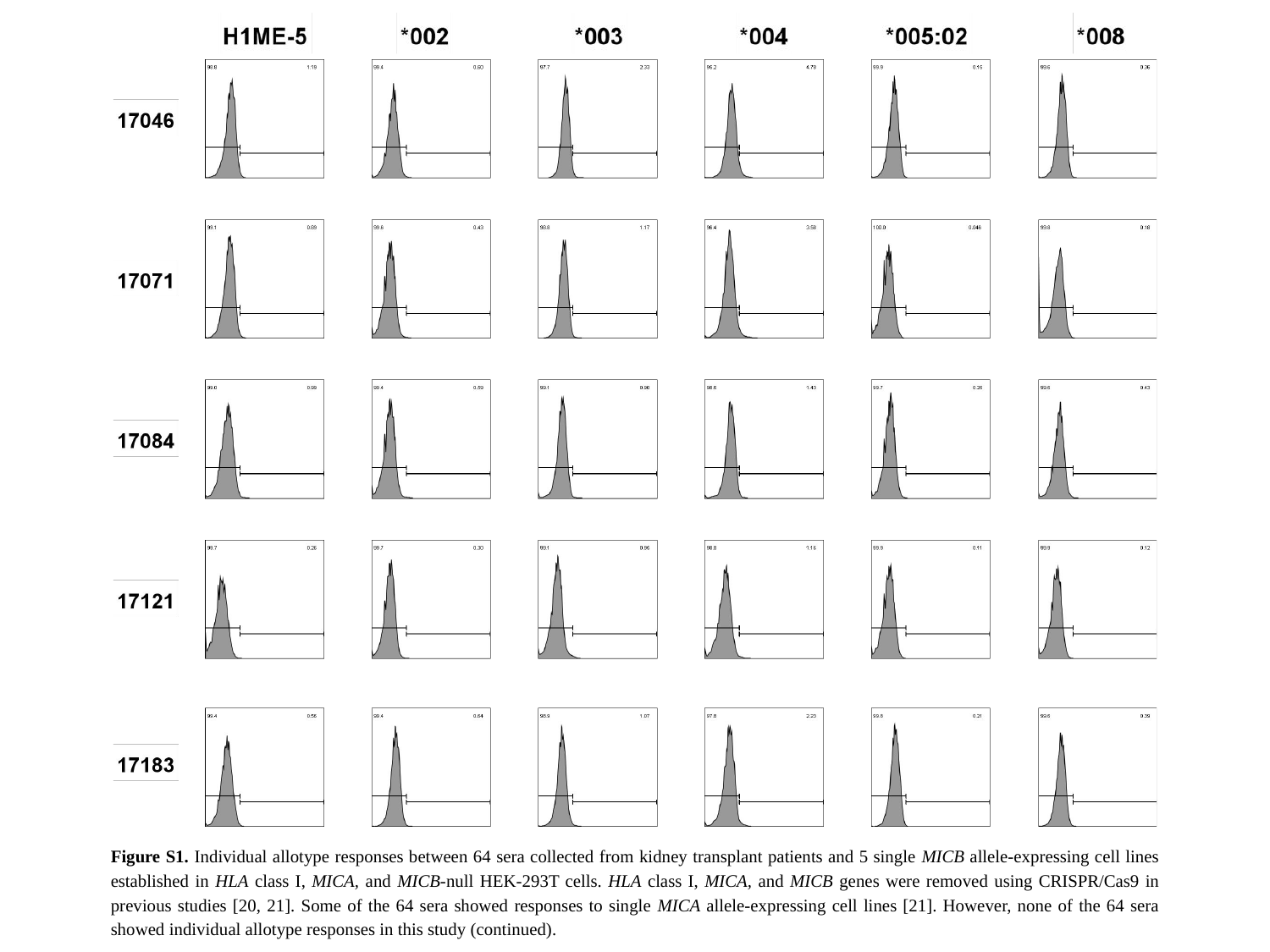

Figure S1. Individual allotype responses between 64 sera collected from kidney transplant patients and 5 single MICB allele-expressing cell lines established in HLA class I, MICA, and MICB-null HEK-293T cells. HLA class I, MICA, and MICB genes were removed using CRISPR/Cas9 in previous studies [20, 21]. Some of the 64 sera showed responses to single MICA allele-expressing cell lines [21]. However, none of the 64 sera showed individual allotype responses in this study (continued).

## Slide 10
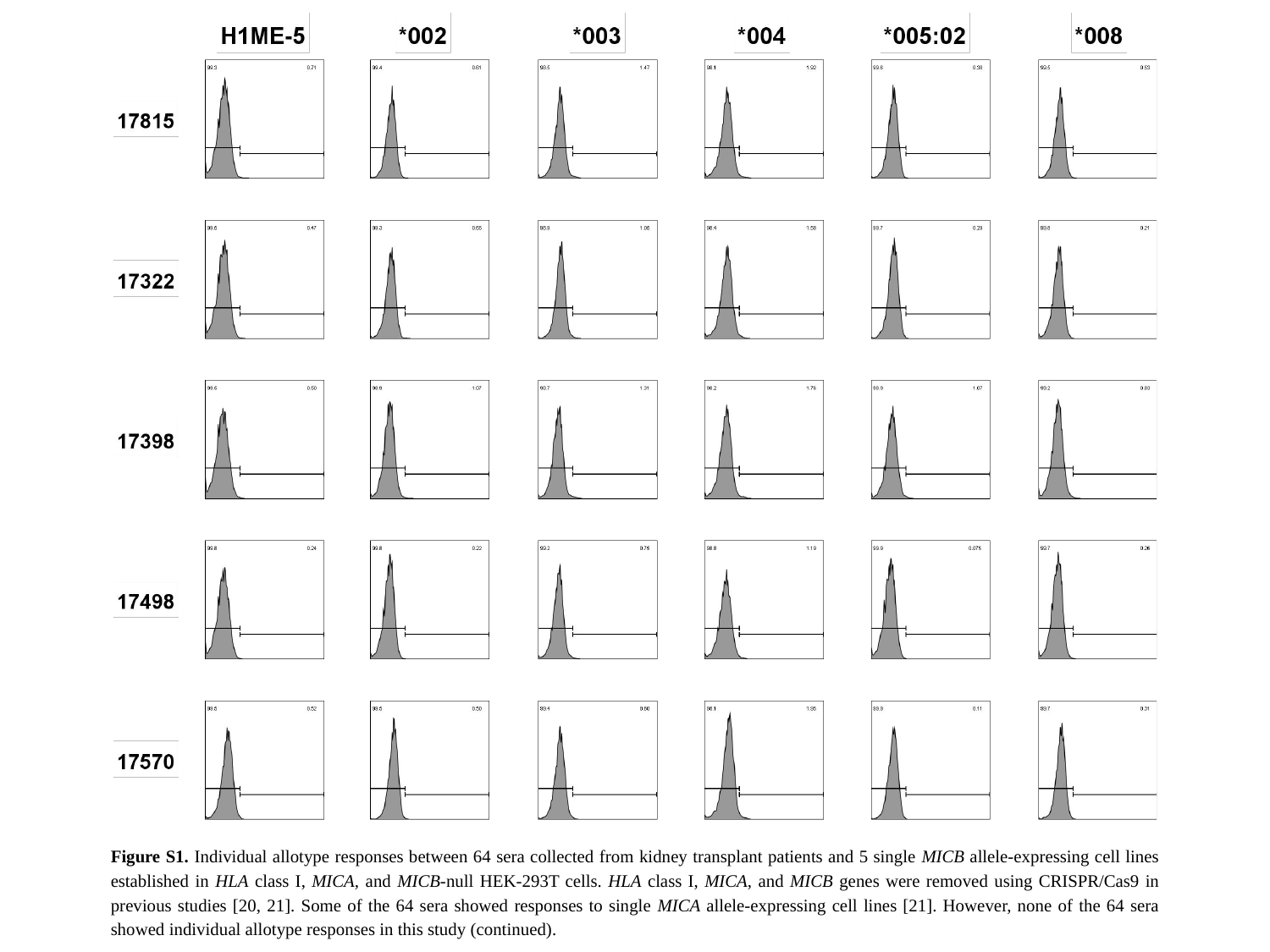

Figure S1. Individual allotype responses between 64 sera collected from kidney transplant patients and 5 single MICB allele-expressing cell lines established in HLA class I, MICA, and MICB-null HEK-293T cells. HLA class I, MICA, and MICB genes were removed using CRISPR/Cas9 in previous studies [20, 21]. Some of the 64 sera showed responses to single MICA allele-expressing cell lines [21]. However, none of the 64 sera showed individual allotype responses in this study (continued).

## Slide 11
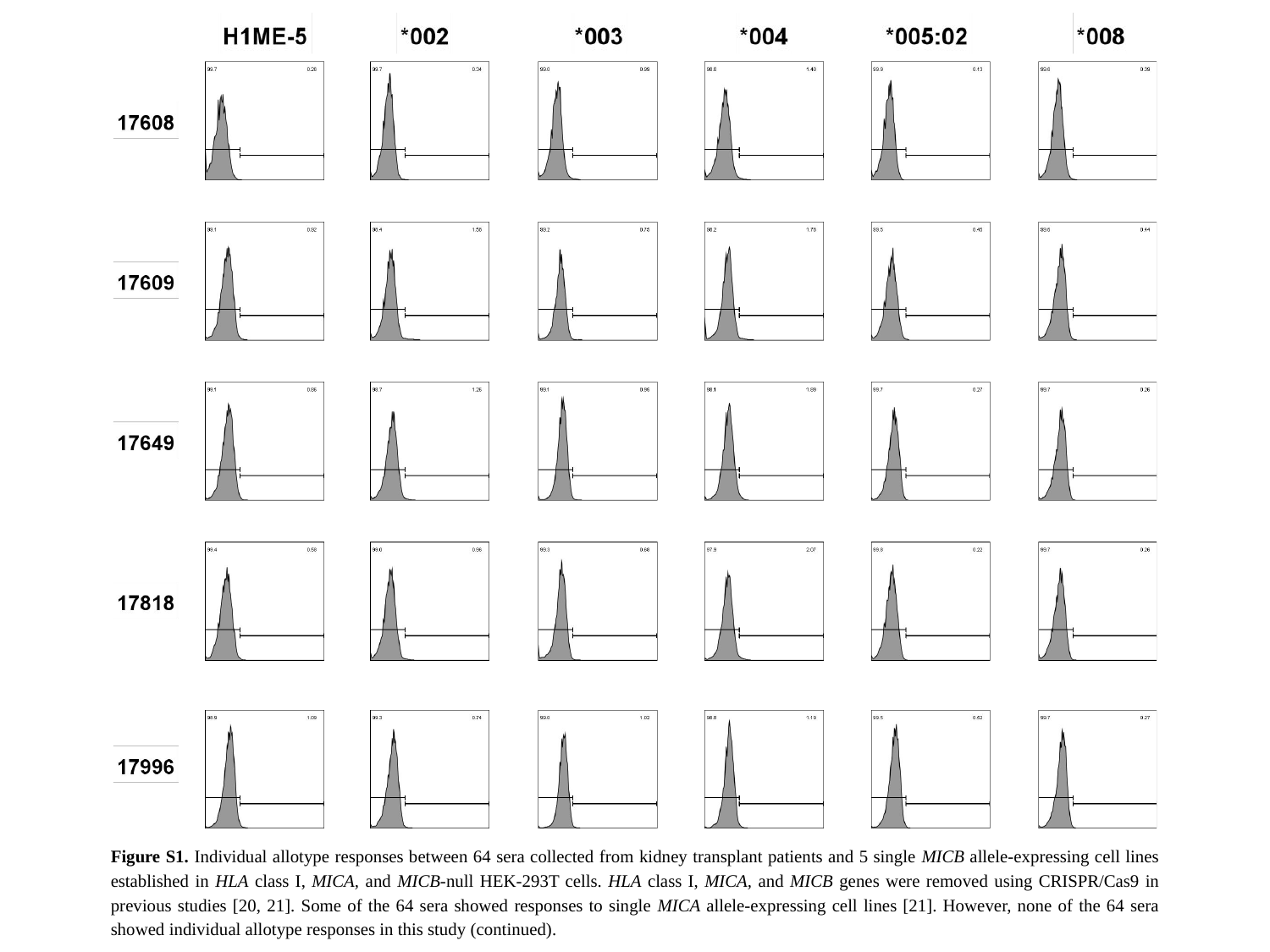

Figure S1. Individual allotype responses between 64 sera collected from kidney transplant patients and 5 single MICB allele-expressing cell lines established in HLA class I, MICA, and MICB-null HEK-293T cells. HLA class I, MICA, and MICB genes were removed using CRISPR/Cas9 in previous studies [20, 21]. Some of the 64 sera showed responses to single MICA allele-expressing cell lines [21]. However, none of the 64 sera showed individual allotype responses in this study (continued).

## Slide 12
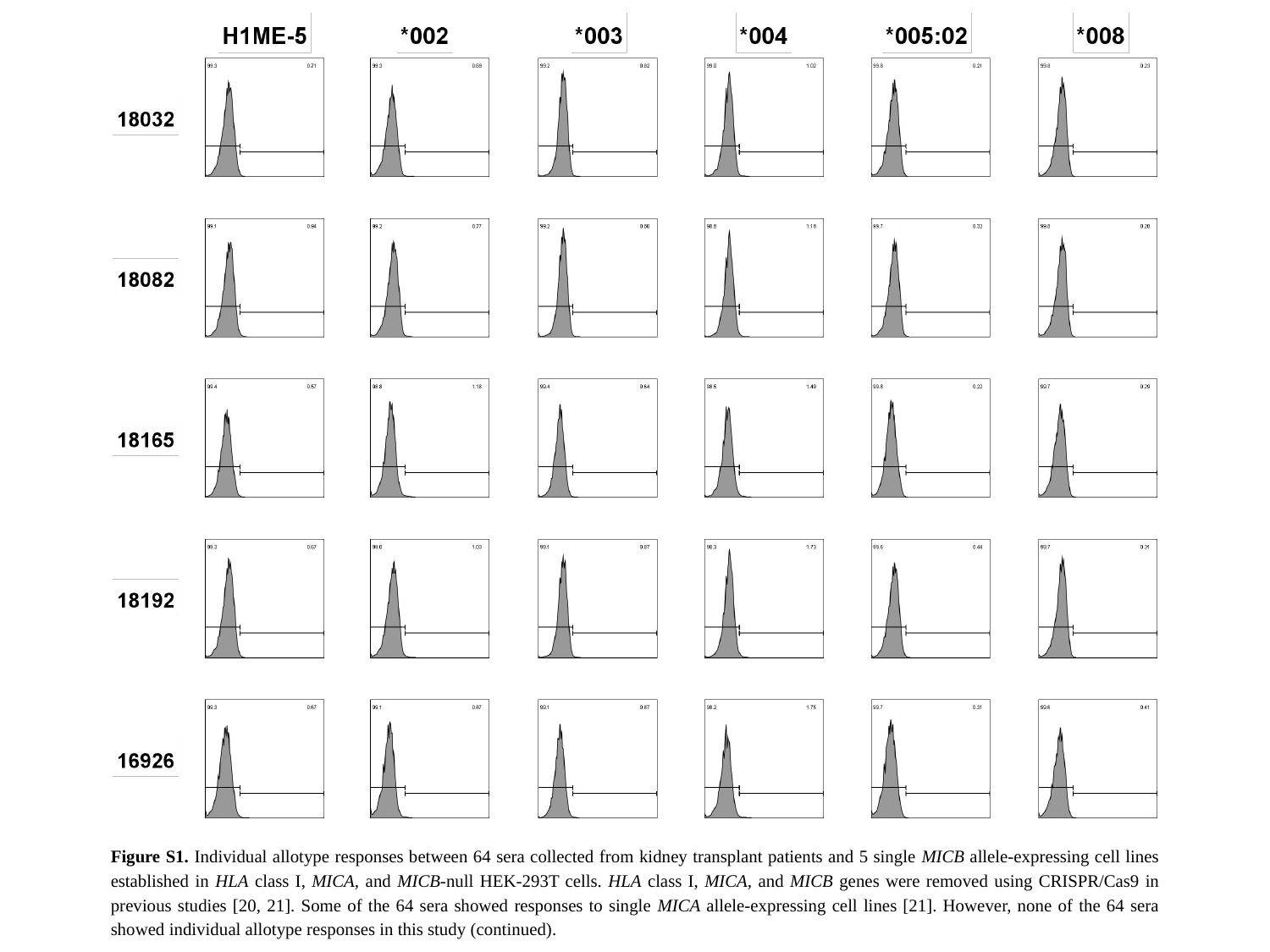

Figure S1. Individual allotype responses between 64 sera collected from kidney transplant patients and 5 single MICB allele-expressing cell lines established in HLA class I, MICA, and MICB-null HEK-293T cells. HLA class I, MICA, and MICB genes were removed using CRISPR/Cas9 in previous studies [20, 21]. Some of the 64 sera showed responses to single MICA allele-expressing cell lines [21]. However, none of the 64 sera showed individual allotype responses in this study (continued).

## Slide 13
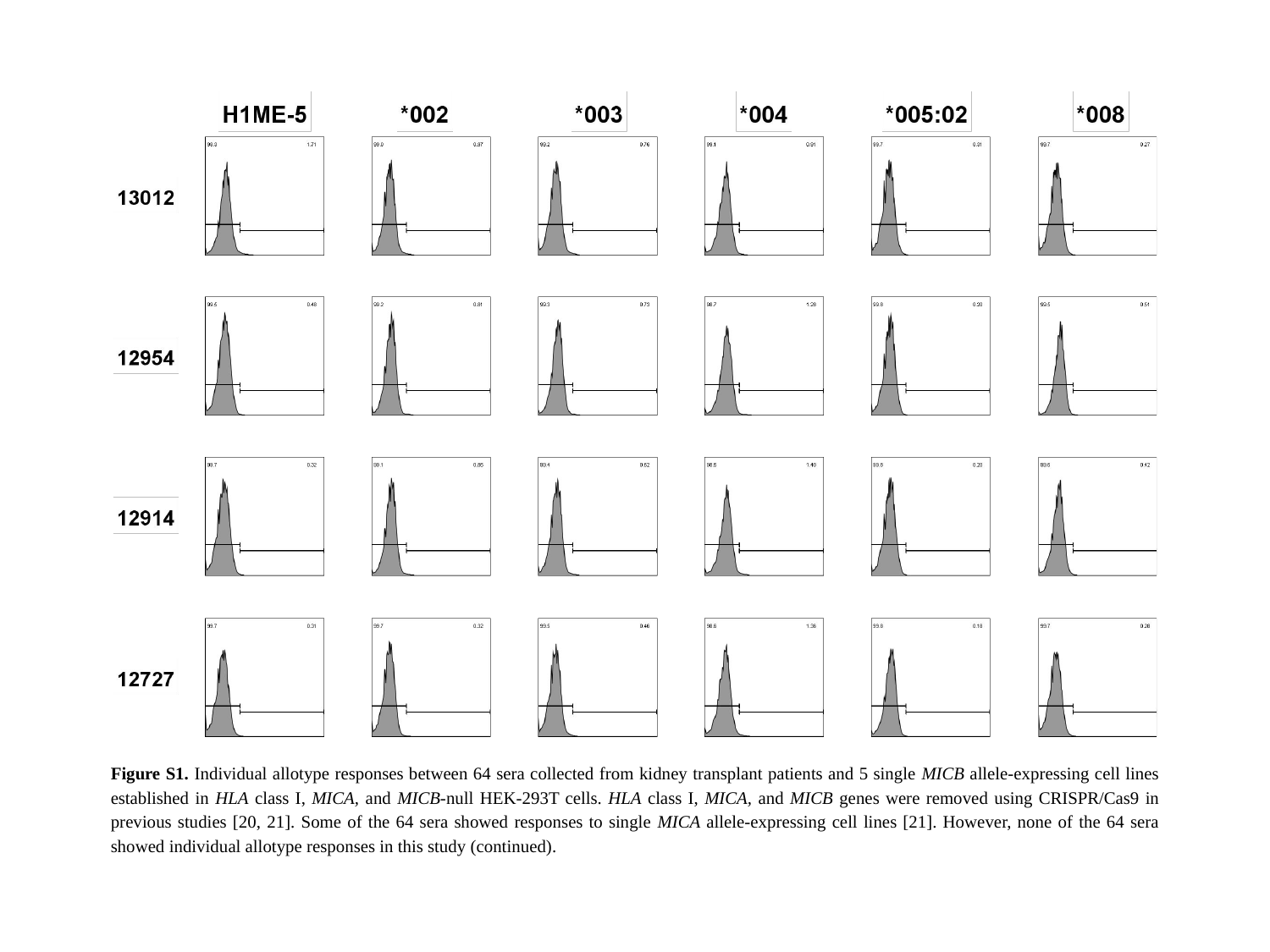

Figure S1. Individual allotype responses between 64 sera collected from kidney transplant patients and 5 single MICB allele-expressing cell lines established in HLA class I, MICA, and MICB-null HEK-293T cells. HLA class I, MICA, and MICB genes were removed using CRISPR/Cas9 in previous studies [20, 21]. Some of the 64 sera showed responses to single MICA allele-expressing cell lines [21]. However, none of the 64 sera showed individual allotype responses in this study (continued).
